# Renal Function in Children with Febrile Convulsions

**Published:** 2014

**Authors:** Ladan AFSHARKHAS, Azita TAVASOLI

**Affiliations:** 1. Pediatric Neurology Department, Ali-Asghar Children’s Hospital, Iran University of Medical Science, Tehran, Iran

**Keywords:** Febrile Convulsion, Electrolyte, UTI

## Abstract

**Objective:**

Febrile convulsions (FC) are the most frequent seizure disorder in children. Some studies have detected serum electrolyte disturbances in patients with FC. This study determines serum electrolytes, renal function tests, and frequency of urinary tract infection in hospitalized children with FC.

**Materials & Methods:**

In this descriptive, cross sectional study, we evaluated 291 children with FC admitted to the Neurology ward of Ali-Asghar Children’s Hospital from 2008– 2013. Data was recorded on age, sex, type (simple, complex), and recurrence of seizures, family history of FC and epilepsy, serum electrolytes, renal function tests, and urinary tract infections.

**Results:**

A total of 291 patients with diagnosis of FC were admitted to our center. Of these 291 patients, 181 (62.2%) were male. The mean age was 24.4 ± 14.6 months. There were simple, complex, and recurrent FCs in 215 (73.9%), 76 (26.1%) and 61 (21%) of patients, respectively. Urinary tract infections (UTI) were found in 13 (4.5%) patients, more present in females (p-value = 0.03) and under 12 months of age (p-value = 0.003). Hyponatremia, hypocalcemia, and hypokalemia was detected in 32 (11%), 16 (5.5%), and 4 (1.4%) of cases, respectively. Twentyfour (8.2%) patients had a glomerular filtration rate less than 60 ml/min/1.73m2.

There were no abnormalities in serum magnesium, BUN, and creatinine levels.

**Conclusion:**

During FCs, mild changes may occur in renal function but a serum electrolyte evaluation is not necessary unless patients are dehydrated. In children with FC, urinary tract infections should be ruled out.

## Introduction

Febrile convulsions (FC) are the most common seizure disorder in children 6–60 months of age with peak incidence at 18 months (1,2). FC affects 2–14% of children and every pediatrician must deal with this disorder (1,3).The International League against Epilepsy (ILAE) has defined a febrile seizure as “a seizure which was associated with a febrile illness in the absence of a CNS infection or an acute electrolyte imbalance in children who were older than 1 month of age, who did not have prior afebrile seizures” (4). The pathogenesis of FC is multifactorial. Familial studies have detected an occurrence rate from 10–46% and median a recurrence rate of 36% in children with a positive familial history for this disorder. The genetic background of FC involves individual and familial susceptibility, modulation of immune response, and neuronal excitability and interactions with exogenous agents such as viruses (5). Primary importance in the diagnostic assessment of children with FC is finding the cause of the fever. It is essential to exclude underlying meningitis in all children with febrile seizures either clinically or by lumbar puncture (6). There are studies that have detected disturbances of serum electrolytes and trace elements such as calcium, sodium, magnesium, iron, zinc, and copper in children with FC (2, 7, 8, 9, 10). However, in absence of positive findings in a patient history such as vomiting and diarrhea or signs of dehydration on physical examination, a determination of serum electrolytes may have limited value. It is worth mentioning that one of the important causes of infection in children with FC is urinary tract infection (UTI). UTI may present as a simple cystitis or pyelonephritis. The signs and symptoms of UTI in children are different and depend on age. Some studies have suggested that every child with FC to be evaluated for UTI by performing urinalysis and urine culture tests, because of a relatively high frequency of UTI among children with the disorder (2–9%) (11, 12). It is notable that during acute febrile disease, production of albumin in the liver decreases, the nitrogen balance begins to be decrease soon after the onset of fever, the blood pressure rises, and glomerular filtration rate (GFR) decreases. Proteinuria occurs in 5–10% of children with fever without pre-existing renal diseases (13). The present study determines serum electrolytes, blood urea nitrogen (BUN), creatinine, GFR, and UTI prevalence in patients with FC.

## Materials & Methods

Our study is a retrospective, cross sectional study on children who were admitted with FC in Ali-Asghar Children’s Hospital, which is a tertiary training medical center- in Tehran, Iran, from December 2008–December 2013. The project was approved by the ethical committees of the Iran University of Medical Sciences. We enrolled 291 patients in an age group of 6-months to 5-years with febrile seizure. Patients with a history of illnesses that effect serum electrolytes and renal function including dehydration, meningitis or encephalitis, and renal or endocrine diseases were excluded. Collected data included age, gender, type of convulsions (simple or complex), recurrence of seizures (more than one time FC during the life of the patient), family history of FC and epilepsy in first and second relatives. Other data related to kidney function included serum levels of sodium (Na < 135meq/L as hyponatremia), potassium (K ≤ 3.5meq/L as hypokalemia), calcium (Ca < 8.5mg/ dl as hypocalcemia), magnesium (Mg < 1.3mg/dl as hypomagnesaemia), blood urea nitrogen (normal range of 10–30 mg/dl), creatinine (normal range of 0.3–0.8 mg/dl), GFR greater than 60 ml/min/1.73m^2 ^were all normal values. The glomerular filtration rate was calculated with the Schwartz formula (GFR = K ×height/serum creatinine, in which K is a constant that depends on muscle mass and itself varies with age; in the first year of life, for pre-term babies K = 0.33 and for full-term infants K = 0.45; for infants and children 1–12 years of age K = 0.55) (11). The diagnosis of UTI was based on clinical manifestation, abnormal urine analysis, and positive urine cultures (a colony count of microorganisms greater than 105 in the high power field) after nephrological consultation. It is worth mentioning that simple FC is a generalized febrile seizure that occurs just one time in 24-hours with a duration of less than 15 minutes and complex FC is a febrile seizure that has focal pattern, lasts longer than 15 minutes, or occurs more than one time in a period of 24 hours (2). 

All data obtained from medical records and collected in questionnaires. Statistical analysis was performed using SPSS for windows (ver. 16). We employed the student’s t-test for comparing variables in two groups and a Pearson correlation for association between variables. P-values of less than 0.05 were considered significant.

## Results

Over a period of five years, 291 patients with FC were admitted to our center. Of the 291 patients, 181 (62.2%) were male. The mean age was 24.4 ± 14.6 months. A total of 215 (73.9%) patients had simple FC and 76 (26.1%) patients had complex seizures. A total of 61 (21%) of patients had recurrent seizures. There was a positive family history of FC and epilepsy in 86 (29.6%) and 38 (13.1%) of cases, respectively. UTI was seen in 13 (4.5%) of patients with frequency of 3.5% in females and 1% in males. Moreover, UTI was prevalent more in females than in males (p-value = 0.003). Patients with UTI had a mean age of 17.7 ± 13.01 and the majority of patients (7 cases) were in the range of 6–12 months (p-value = 0.03). Five cases of patients had positive urine culture, 3 cases indicated E.coli, and 2 cases indicated pseudomona aeruginosa. Table 1 depicts the mean serum levels of sodium, potassium, calcium, magnesium, BUN, creatinine, and GFR. Hyponatremia in 32 (11%), hypocalcemia in 16 (5.5%), and hypokalemia in 4 (1.4%) cases was detected. GFR was under 60 ml/min/1.73m2 in 24 (8.2%) patients. Abnormal findings in renal function tests of children with simple,complex and recurrent FC are compared in table 2.Hypokalemia was prevalent more in patients with complex FC(p-value=0.02). There were no abnormalities in serum magnesium, BUN, and creatinine levels. Statistical analysis was performed using SPSS (ver. 16). We performed a Student’s t-test for comparing variables in the two groups and a Pearson correlation for the association between variables. P-values of less than 0.05 were considered significant.

## Discussion

Febrile Seizures are common in childhood and many physicians do extend the investigation for these benign events. There are some reports that suggest no routine laboratory examinations i.e., serum electrolytes are needed for patients with simple FC (3, 7). During acute febrile disease, arginine vasopressin (AVP) is an endogenous antipyretic that is secreted from pituitary gland in an attempt to control fever. AVP is also responsible for the maintenance of homeostasis of body fluids during fever. Hyponatremia often occurs in association with acute febrile diseases, particularly with pneumonia and meningitis, because of inappropriate secretions of AVP. These disturbances might enhance risk for seizure and their recurrence during a febrile period (13). In our study, a few patients had abnormal levels of serum sodium (32 cases), potassium (4 cases), and calcium (16cases) levels but there were no significant differences between patients with simple, complex, and recurrent febrile convulsions (Table -2). Similar to our study, Heydarian et al observed no significant differences in the serum sodium levels between the simple and recurrent febrile seizure groups (14). Karimzadeh et al detected that among 289 patients with FC, there were hyponatremia, hypokalemia, and hypocalcemia in nine, one, and one cases, respectively. This suggested that most of the paraclinical examinations are unnecessary for children with FC, especially for simple FC (7). 

Nikavar et al reported that there was no significant difference between serum sodium and calcium levels of children with simple and recurrent FC and suggested routine serum electrolyte screening is not recommended for febrile seizures (15). Some studies have shown decreased serum levels of neurotransmitters and trace elements such as magnesium contribute as a risk factor of FC (16). However, in our study, serum levels of magnesium were 2.2 ± .293 mg/dl and there were no cases of hypomagnesaemia in patients with simple, complex, and recurrent FC. This difference may be due to different measuring techniques and lack of a control group (patients without seizures as the control group) in our assessment. In our survey, although mean GFR were in the normal range, there were no differences between simple, complex, and recurrent FC cases. A total of 24 patients had a GFR under normal limits. This could be attributed to fever and suggested that the initial phase of fever is accompanied by a rise in blood pressure and a decrease in GFR. A sustained fever is associated with a fall in blood pressure and a slight increase in GFR (13). 

The sources of infection in children with FC vary and include upper respiratory tract infections, otitis media, pneumonia, gastroenteritis, and UTI that presented as a simple cystitis or pyelonephritis (12, 17). In our study, the frequency of UTI was 4.5% with a higher rate in females and patients under 1 year of age. This confirms the results of Momen et al. They found that the frequency of UTI among children with FC was 6.6% with a higher incidence of UTI in females than in males (3–5% and 1%, respectively). They recommended that UTI should be considered as an important cause of FC in children (16).

Our study had some limitations such as its retrospective nature and the lack of control group of patients with fever without seizure for evaluating the effects of seizure on renal function.

Based on our findings, during FC, mild changes may occur in renal function. We suggest that renal evaluation tests especially serum electrolytes are not necessary to be done as routine laboratory examinations for children with FC unless patients present with gastroenteritis, vomiting, or presence of signs and symptoms of dehydration. It is recommended in any patient with FC, that urine analysis be done to exclude UTI. 

**Table 1:** Results of Serum Levels of Electrolyte and Kidney Function Tests

Tests	Na(meq/l)	K(meq/l)	Ca(mg/dl)	Mg(mg/dl)	BUN(mg/dl)	Cr(mg/dl)	GFR (ml/min/1.73m^2^)
Mean Standard deviation	138.699 14.644	4.428 0.608	9.340 0.677	2.2 0.293	10.742 3.963	.515 0.122	86.904 23

**Table 2:** Types of FC and Abnormal Findings in Renal Function Tests

**Abnormal renal tests**	**Simple FC** **No(%)**	**Complex FC** **No(%)**	**P- Value**	**Recurrent FC** **No(%)**	**p-value**
Hyponatremia	22(10.2%)	10(13.1%)	>0.05	8(13.1%)	>0.05
Hypokalemia	1(0.47%)	3(4%)	0.02	0(0%)	>0.05
Hypocalcemia	12(5.6%)	4(5.3%)	>0.05	3(4.9%)	>0.05
decreased GFR	17(7.9%)	7(9.2%)	>0.05	5(8.1%)	>0.05
Urinary Tract Infection	10(4.7%)	3(4%)	>0.05	2(3.3%)	>0.05
